# Risk factors of osteoporosis in elderly inpatients: A cross-sectional single-centre study

**DOI:** 10.3389/fragi.2023.1126172

**Published:** 2023-05-09

**Authors:** Han Li, Tianbao Sun, Dongmei Han, Weiwei Gong, Weiwei Mao, Xianze Gan, Dan Shu, Qian Zhou, Lei Xu, Liufang Hou, Mingcheng Zhou, Mingwei Cai, Xueli Lai

**Affiliations:** ^1^ The Rehabilitation Department of Nephrology, The First Rehabilitation Hospital of Shanghai, Shanghai, China; ^2^ School of Medicine, Tongji University, Shanghai, China; ^3^ The Rehabilitation Department, The First Rehabilitation Hospital of Shanghai, Shanghai, China; ^4^ The Rehabilitation Department of Orthopedics, The First Rehabilitation Hospital of Shanghai, Shanghai, China; ^5^ Department of Nephrology, Shanghai Changhai Hospital, Shanghai, China

**Keywords:** elderly, bone mineral density, osteoporosis, nutrition, exercise capacity, uric acid

## Abstract

**Objective:** This study aimed to identify factors significantly associated with the occurrence of osteoporosis in elderly and very elderly patients.

**Methods:** Elderly hospitalized patients who were older than 60 years old, from the Rehabilitation Hospital from December 2019 to December 2020 were selected. Barthel index (BI), nutritional assessment, the causes of bone mineral density (BMD) reductions in elderly and elderly patients were analysed.

**Results:** A total of 94 patients (83.56 ± 8.37 years old) were enrolled. With increasing age, the BMD of the lumbar spine, femoral neck, and femoral shaft of elderly patients significantly decreased, and the incidence of osteoporosis (OP) significantly increased. The BMD of the lumbar spine was negatively correlated with female and positively correlated with serum 25-hydroxyvitamin D levels, the difference between actual body weight and ideal body weight, and blood uric acid levels; The BMD of the femoral neck was negatively correlated with age and female, and positively correlated with height and geriatric nutrition risk index score. The BMD of the femoral shaft was negatively correlated with female and positively correlated with BI.

**Conclusion:** With increasing age, the BMD of the lumbar spine and the femoral shaft significantly decreased, and the incidence of OP significantly increased in elderly and very elderly patients. Aric acid may protect bone health in elderly patients. Early attention to the nutritional status, exercise capacity, 25-hydroxyvitamin D level, and blood uric acid level in the elderly population can help identify high-risk elderly patients with OP.

## 1 Introduction

Population ageing has become a public health problem worldwide. Numerous studies have shown that by 2050, the growth rate of the population over 60 years old in the world will reach 21%, and the total population will reach 2.5 billion (Beard et al., 2016; He et al., 2016). Compared with Japan, India and even European and American countries, China has the highest proportion of elderly people in the world. With the ageing of the population, the decline in nutritional status, the slowness of movements, and the loss of exercise capacity will cause a series of complications, such as muscle atrophy and osteoporosis (OP), which will further cause a decline in exercise capacity and balance and eventually lead to an increased risk of falls and an increased risk of fractures in elderly individuals (Schaap et al., 2018).

OP is the most common bone disease and is a systemic bone disease characterized by low bone mass and damage to the microstructure of bone tissue, leading to increased bone fragility and susceptibility to fractures (Rossini et al., 2016). With the ageing of the population, increased bone fragility in OP and related diseases due to the loss of cortical bone and trabecular bone fragments is widely considered the main cause of fractures in the elderly. The latest epidemiological survey of OP showed that the incidence of OP in people aged 60 years and older was 10.7% in males and 51.6% in females (Yoshimura et al., 2017). The incidence of OP is increasing, and it is estimated that there are approximately 200 million OP patients worldwide (Sözen et al., 2017).

As a multifactorial systemic disease, many factors can lead to OP. Poor nutritional status, reduced muscle content, and progressive and extensive loss of muscle mass and function may be associated with reduced bone mineral density (BMD) (Dong et al., 2016). One study reported a 30% prevalence of sarcopenia in people over 60 years of age, and this prevalence will continue to grow with the ageing of the population. The decline in muscle mass and strength in elderly patients may limit their abilities in daily life, thereby increasing OP prevalence and mortality. In addition, malnutrition and reduced intake are also generally considered to be closely related to OP. Scholars suggest that clinical tools can be used to assess nutritional status, for example, general malnutrition screening tools (Torres et al., 2015), mini scale of nutrition, and the geriatric nutrition risk index (GNRI) (Chiu et al., 2020). The GNRI jointly uses the body mass index (BMI) and serum albumin to generate a nutritional index score. In addition, some hormones, such as oestrogen and glucocorticoids, as well as some biochemical markers of bone turnover, such as osteocalcin and 1,25-hydroxyvitamin D, affect the occurrence of OP. From the abovementioned studies, the following conclusions have been obtained: 1) OP is a multifactorial systemic disease; And 2) the evaluation of nutritional status parameters, exercise status parameters and some bone turnover markers in elderly patients can help to identify OP patients for early intervention and treatment. But little is known about risk factors for OP in very elderly patients. Therefore, we conducted this study with the aim of further analysing OP in elderly hospitalized patients on the basis of the above research and identifying the association of age, sex, nutritional risk, serum 25-hydroxyvitamin D, and physical function with bone mineral density in elderly and very elderly patients.

## 2 Materials and methods

### 2.1 Study design and participants

This study included convenient sample, elderly patients ≥60 years of age who were hospitalized at the First Rehabilitation Hospital of Shanghai, China, from December 2019 to December 2020. The exclusion criteria were as follows: patients with a history of excessive alcohol consumption, severe hepatic or renal impairment, diseases that severely affect bone or calcium metabolism, such as abnormal thyroid function and parathyroid function, acute gout, acute infections or acute complications of diabetes, acute heart failure, other malignancies, celiac disease, inflammatory bowel disease, systemic lupus erythematosus, rheumatoid arthritis, multiple myelomas and a history of any organ transplantation. Dietary calcium supplementation was permitted. Patients were informed about the purpose and risks of the study and participated voluntarily. This study was reviewed and approved by the Ethics Committee of the First Rehabilitation Hospital of Shanghai in accordance with the Helsinki Declaration (YK 2018-02-006).

### 2.2 Observation indicators and methods

The history of the study subjects was collected: history of smoking, drinking, underlying diseases, and fractures and anthropometric measurement indicators (height, body weight, BMI, waist circumference, hip circumference, waist-to-hip ratio, and waist skinfold thickness).

The following serological indicators were assessed: serum albumin, serum alkaline phosphatase, cholesterol, serum urea, serum creatinine, serum uric acid, fasting blood glucose, parathyroid hormone, osteocalcin, 25-hydroxyvitamin D, thyroid-stimulating hormone (TSH), serum calcium, and serum phosphorus. All biochemical measurements were performed using standardized methods in the biochemical laboratory of our hospital.

The BMD of the lumbar spine (L2-L4), femoral neck, and femoral shaft was assessed using dual-energy X-ray bone absorptiometry (DXA). All patients were scanned by a radiographer to minimise measurement variation. The T-score was used to compare the peak bone mass of the study subjects with that of sex- and race-matched individuals. OP was diagnosed using the World Health Organization (WHO) (1994) diagnostic criteria: T-score ≤ −2.5, OP; −2.5 < T-score < −1.0, osteopenia; and T-score ≥ −1.0, normal bone mass.

Nutritional assessment indicators:

① Calculation of GNRI: The GNRI was used to assess the nutritional status of high-risk elderly patients and was calculated using the baseline body weight and serum albumin level as follows (Bouillanne et al., 2005): GNRI = 1.489 × serum albumin (g/L) + 41.7 × (body weight/ideal body weight). If the body weight of an individual was greater than the ideal body weight, the body weight/ideal body weight was calculated as 1. Ideal body weight for males = 0.75 × height (cm) - 62.5; ideal body weight for females = 0.60 × height (cm) −40. For patients who had difficulty standing upright and for whom height was unable to be measured, height was estimated by measuring knee height. Height of the males = 2.02 × knee height (cm)—0.04 × age + 64.19; height of the females = 1.83 × knee height (cm)—0.24 × age + 84.88.
② Conicity index (CI) measurement and calculation: The CI was used to assess the risk of nutritional and metabolic diseases in the body (Zheng et al., 2016). As early as the end of the 1990s, the CI has been used to assess obesity, body fat distribution, the presence of centripetal obesity, and the presence of risk factors for obesity in elderly patients. It is also used to assess the nutritional status of adult haemodialysis patients and predict the mortality rate. CI = waist circumference/(0.109 × square root of body weight/height); Exercise capacity scale assessment: activities of daily living (ADL) scale and Karnofsky scale. The ADL (Barthel index) measures the ability of a person to perform daily activities to meet daily behaviour needs (Hsu et al., 2019). The Karnofsky scale is a self-rating instrument used to assess activity status.

### 2.3 Statistical analysis

vStatistical analysis was performed using SPSS 21.0 statistical software. Measurement data with a normal distribution are presented as x ± s, and data with a non-normal distribution are presented as M (1/4, 3/4). Normally distributed data were compared using independent sample t-tests, non-normally distributed data were compared using non-parametric tests, and count data were compared using chi-squared tests. Pearson correlation analysis was used for normally distributed data, and Spearman correlation analysis was used for non-normally distributed data. Variables that were significantly different in the univariate analysis and related variables were included in the multivariate stepwise regression model to analyse independent related factors. *p* < 0.05 was considered statistically significant.

## 3 Results

### 3.1 A total of 94 patients (32 males and 62 females, aged 63–98 years at baseline)

Were enrolled in this study and completed all the test items (Table 1).

① General and relevant information: Seven patients had a history of alcohol consumption, and 15 patients had a history of smoking. Seventy-five patients had a history of hypertension, and 45 patients had a history of diabetes. Twenty patients had a history of fractures.
② DXA results: A total of 11 patients had normal bone mass, including 1 patient with a history of fractures, and 24 patients had reduced bone mass, accounting for 25.5% of the study population, including 3 patients with a history of fractures. Fifty-nine patients had OP, accounting for 62.8%, of whom 16 had a history of fractures.
③ Nutritional status results: The average BMI was 22.59 ± 3.67 kg/m2, the GNRI score was 84.88 (87.55, 94.35), and the CI was 0.40 (0.37, 0.41).
④ Exercise capacity results: The average ADL score was 48.83 ± 25.64, and the average Karnofsky score was 50.77 ± 12.52.

**TABLE 1:** Baseline and DXA characteristics.

Characteristics	All patients
	(*n* = 94)
Age (year)	83.56 ± 8.37
Sex (men, %)	62 (66.0)
Height(m)	1.63 ± 0.09
Weight (kg)	61.55 ± 10.35
BMI (kg/m2)	22.59 ± 3.67
Waist hip rate	0.87 ± 0.07
Waistline m) [M(1/4, 3/4)]	0.81 (0.73, 0.86)
Weight difference (actual weight-ideal weight) (1/4, 3/4) (kg)	2.00 (−1.61, 7.00)
GNRI (score) (1/4, 3/4)	84.88 (87.55, 94.35)
CI(1/4, 3/4)	0.40 (0.37, 0.41)
Laboratory parameters	33.0 (29.00, 35.50)
Albumin (g/dL) [M(1/4, 3/4)]	7.18 ± 4.40
BUN(mmol/L)	323.61 ± 126.85
UA (umol/L)	77.23 ± 64.87
SCr(mmol/L)	2.08 (2.01, 2.18)
Total calcium (mg/dL) [M(1/4, 3/4)]	1.05 ± 0.20
P (mmol/L)	47.98 ± 45.29
PTH (pg/mL)	20.77 ± 13.63
Osteocalcin (mmol/L)	9.01 ± 5.51
Vit D (ng/mL)	91 (96.8%)
<20 ng/mL	2.76 ± 1.71
TSH (ng/mL)	3.44 (2.87, 4.08)
Cholesterol (mmol/L) [M(1/4, 3/4)]	5.63 ± 1.65
Fasting blood glucose (mmol/L)	69.84 ± 22.0
Alkaline phosphatase (ALP) (mmol/L)	
DXA	11 (11.7%)
Normal bone mass (case, %)	1
History of fracture (case)	24 (25.5%)
Osteopenia (case, %)	3
History of fracture (case)	59 (62.8%)
Osteoporosis (case, %)	16
History of fracture (case)	
DXA Parameters	0.94 ± 0.23
Lumbar spine (L1-4)BMD (g/cm2)	−1.90 (−2.75, 0.00)
T score [M(1/4, 3/4)]	0.60 ± 0.24
Femoral neck BMD (g/cm2)	−2.70 (−3.50, −1.60)
T score [M(1/4, 3/4)]	0.65 ± 0.28
Total femoral shaft BMD (g/cm2)	−2.41 ± 2.11
T score [M(1/4, 3/4)]	
Exercise capacity	48.83 ± 25.64
ADL score	50.77 ± 12.52
Karnofsky score	

Abbreviations: GNRI, geriatric nutrition risk index; BMI, body mass index; DXA, dual-energy X-ray absorptiometry; BMD, bone mineral density; TSH, Thyroid-stimulating hormone; PTH, parathyroid hormone; ALP, Alkaline Phosphatase.

### 3.2 The BMD in and T-score for hospitalized elderly patients decreased with increasing age

**FIGURE 1:**
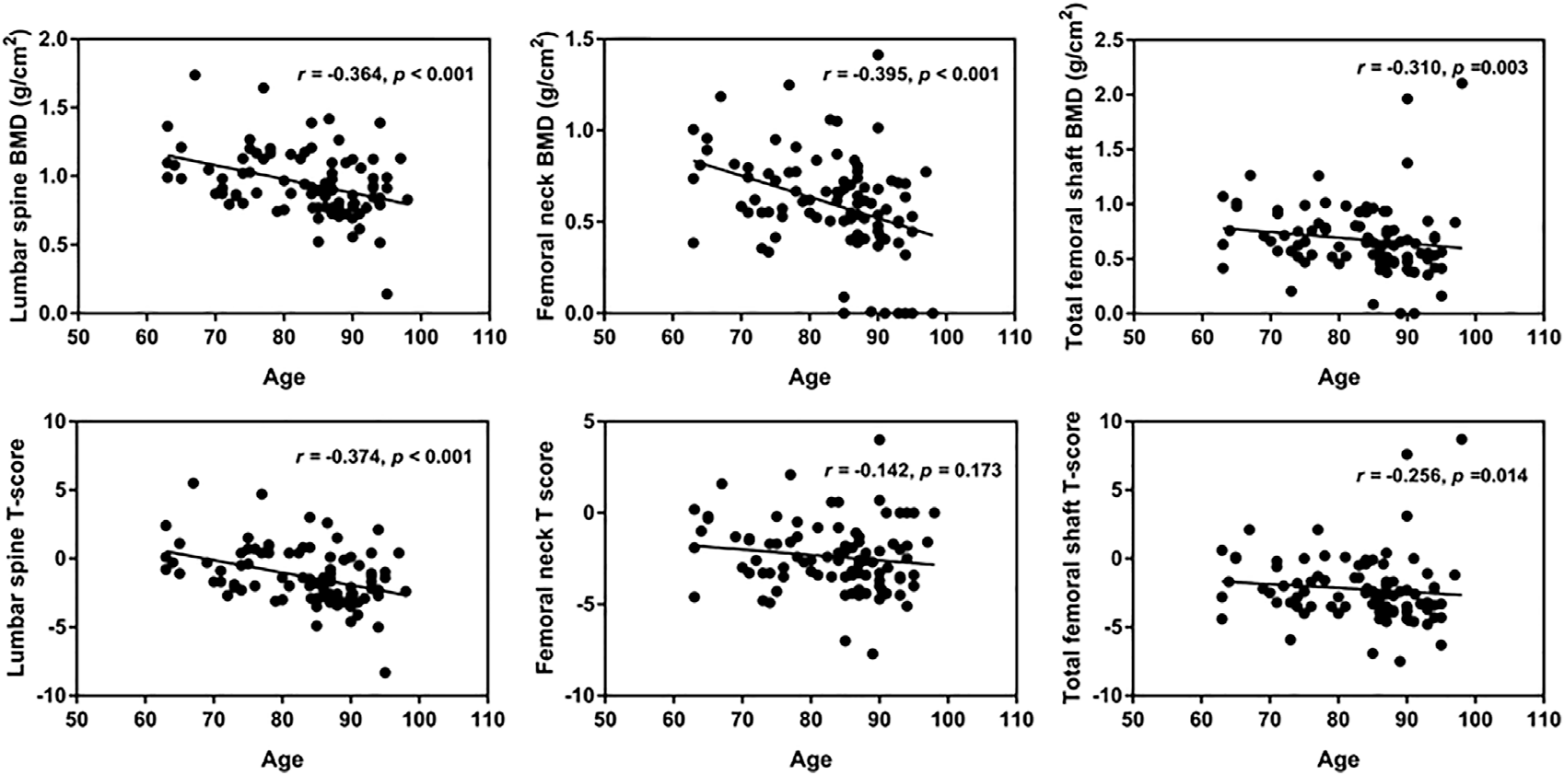
The relationship of lumbar spine BMD and age.

The BMD of the lumbar spine and femoral neck decreased more significantly than that of the femoral shaft, while T-score changes were most significant in the lumbar spine (Figure 1).

### 3.3 Multivariate regression analysis based on BMD

Higher vitamin D level (per mg/mL, log per 1 ng/mL, unstandardized β = 0.007; 95% CI, 0.001 to 0.012; *p* = 0.025), higher body weight, i.e., larger difference between the actual body weight and ideal body weight (unstandardized β = 0.01; 95% CI, 0.004 to 0.016; *p* = 0.001), and higher uric acid level (per nmol/L, log per 1 nmol/L, unstandardized β = 0.000; 95% CI, 0.000 to 0.001; *p* < 0.001) were associated with high lumbar spine BMD; female sex (unstandardized β = −0.234; 95% CI, −0.320 to-0.148; *p* < 0.001) was associated with low lumbar spine BMD. Taller height (unstandardized β = 0.855; 95% CI, 0.244 to 1.466; *p* = 0.027) and higher GNRI (per 1 point; unstandardized β = 0.011; 95% CI, 0.004 to 0.016; *p* = 0.002) were associated with high femoral neck BMD, and age (every 1 year; unstandardized β = −0.008; 95% CI, −0.014 to −0.002; *p* = 0.015) was associated with low femoral neck BMD. Higher ADL index score (per 1 point; unstandardized coefficient β = 0.003; 95% CI, 0.000 to 0.005; *p* = 0.030) was associated with high femoral shaft BMD; female sex (unstandardized β = −0.190; 95% CI, −0.316 to −0.063; *p* = 0.004) was associated with low femoral shaft BMD.

The specific analysis results are provided in Table 2.

**TABLE 2:** Determinants of bone mineral density (BMD) using multivariable stepwise linear regression analysis.

	Multivariate (stepwise)		P	
BMD	Unstandardized coefficient			
	β (95% CI)			
Lumbar spine BMD	−0.234 (−0.320, −0.148)		<0.001	
Sex (Female)	0.007 (0.001, 0.012)		0.025	
Vit D (per ng/mL)	0.010 (0.004, 0.016)		0.001	
Weight difference (kg)	0.000 (0.000, 0.001)		<0.001	
Uric acid (per nmol/L)				
Femoral neck BMD	0.855 (0.244, 1.466)		0.007	
Height (m)	0.011 (0.004, 0.017)		0.002	
GNRI	−0.008 (−0.014, −0.002)		0.015	
Age (per 1 year)				
Total femoral shaft BMD	−0.190 (−0.316, −0.063)		0.004	
Sex (Female)	0.003 (0.000, 0.005)		0.030	
ADL				

BMD, bone mineral density; ADL, activities of daily living; GNRI, Geriatric Nutritional Risk Index.

### 3.4 Multivariate regression analysis based on the T-score

The results of multivariate regression analysis of lumbar spine T-score were basically consistent with the results for lumbar spine BMD. Higher vitamin D level (per mg/mL, log per 1 ng/mL, unstandardized β = 0.056; 95% CI, 0.008 to 0.104; *p* = 0.024), higher body weight, i.e., larger difference between the actual body weight and ideal body weight (unstandardized β = 0.084; 95% CI, 0.034 to 0.134; *p* = 0.001), and higher uric acid level (per nmol/L, log per 1 nmol/L, unstandardized β = 0.004; 95% CI, 0.001 to 0.006; *p* = 0.001 0.01) were associated with high lumbar spine BMD; female sex (unstandardized β = −2.245; 95% CI, −2.967 to −1.522; *p* < 0.001) was associated with low lumbar spine BMD. Higher blood uric acid level (per 1 point, unstandardized β = 0.003; 95% CI, 0.000 to 0.006; *p* = 0.040) was associated with high femoral neck T-score; females (unstandardized coefficient β = −1.336; 95% CI, −2.084 to −0.588; *p* = 0.001) was associated with low femoral neck BMD. Higher ADL index (per 1 point; unstandardized coefficient β = 0.022; the 95% CI, 0.004 to 0.040; *p* = 0.015) was associated with high femoral shaft BMD. Female sex (unstandardized β = −0.120; 95% CI, −2.156 to −0.243) was associated with low femoral shaft BMD.

The specific analysis results are provided in Table 3.

**TABLE 3:** Determinants of T-score using multivariable stepwise linear regression analysis.

	Multivariate (stepwise)	P
T-score	Unstandardized coefficient	
	β (95% CI)	
Lumbar spine T-score		
Sex (Female)	−2.245 (−2.967, −1.522)	<0.001
Vit D (per 1 ng/mL)	0.056 (0.008, 0.104)	0.024
Weight difference (kg)	0.084 (0.034, 0.134)	0.001
Uric acid (per nmol/L)	0.004 (0.001, 0.006)	0.010
Femoral neck T-score		
Sex (Female)	−1.336 (−2.084, −0.588)	0.001
Uric acid	0.003 (0.000, 0.006)	0.040
Total femoral shaft T-score		
Sex (Female)	−1.200 (−2.156, −0.243)	0.015
ADL	0.022 (0.004, 0.040)	0.015

## 4 Discussion

Osteoporosis has become a global public health threat for elderly individuals (He et al., 2016). However, little is known about OP in very elderly patients. The mean age of 83.56 years in our study is much older than that in other studies (Oka et al., 2018; Moriwaki et al., 2019). In this way, the prevalence of OP in hospitalized elderly patients over 60 years in our study was 62.8%, and the prevalence of bone loss was 25.5%, values that are much higher than the epidemiological data for the elderly population. In this study, we also found that elderly patients maintained a state close to the ideal body weight or slightly higher than the ideal body weight, maintained a normal blood uric acid level, and had a good nutritional status; that is, a high GNRI, ability to exercise, and a higher ADL score were all conducive to maintaining high BMD and reducing the occurrence of OP. We recommend using the GNRI to assess the nutritional status of elderly patients. Compared with the CI, the GNRI uses not only height and body weight but also serum albumin level, better reflecting the nutritional status of elderly patients. Compared with the Karnofsky score, the exercise ability score (ADL) can better assess the exercise capacity and self-care ability of elderly patients.

It is found in our study that the serum uric acid level was positively correlated with the BMD of and T-scores for the lumbar spine and femoral neck in elderly patients. Available evidence linking serum uric acid (SUA) and BMD remains controversial. In general, the results from basic experimental studies do not support the protective effect of uric acid on bone health, but there are a few studies that do (Ahn et al., 2013). showed that uric acid reduced osteoclast production in a dose-dependent manner and reduced the production of reactive oxygen species by osteoclast precursors. Another study constructed a rat model of mild hyperuricaemia; BMD and bone biomechanical properties in model rats improved compared with those in control rats with normal uric acid levels (Cosman et al., 2014). Epidemiological studies of the relationship between uric acid and BMD are also inconsistent. Some recent studies have shown that uric acid plays a protective role in bone health (Dong et al., 2016). High uric acid levels are associated with high BMD and a low fracture risk (Ahn et al., 2013). Studies with patients with type 2 diabetes, pre- and post-menopausal women and community populations have shown a significant positive association between uric acid and BMD (Lee et al., 2015; Zhao et al., 2016), and high-normal blood uric acid levels have been shown to help stabilize bone mass. However, some studies have reached opposite conclusions (Bhupathiraju et al., 2007). Some scholars propose that high uric acid levels may aggravate the progression of diabetes or aggravate the reduction in glucose metabolism-related bone mass caused by hyperglycaemia (Zhang et al., 2015). The inconsistent study results may be due to differences in the study population and research methods. The elderly patients included in this study had a blood uric acid level of 323.61 ± 126.85 μmol/L, and there were no patients with excessively high blood uric acid levels or acute gout attacks. Our study also showed a positive correlation between blood uric acid levels and the BMD of the lumbar spine and femoral neck. Uric acid is an end product of purine metabolism in the human body and an important antioxidant substance in the body. It is an independent risk factor for many diseases, such as heart and cerebrovascular diseases and gout arthritis. An increasing number of studies have shown that uric acid plays an important role in antioxidative stress and participates in more than half of the free radical scavenging activities in the human body (Sharma et al., 2018). Therefore, uric acid may protect bone health through antioxidative effects. In addition, uric acid also reflects the nutritional status of the population itself. High uric acid levels indicate a relatively high nutritional status. Therefore, we believe that maintaining the blood uric acid levels in normal range or high range has a protective effect on BMD in elderly patients.

We also found there was a positive correlation between GNRI and BMD in the elderly hospitalized population. Due to the lack of a gold standard for assessing the nutritional status of elderly patients, this study selected an elderly nutritional risk index (GNRI) and the CI as references to reflect the nutritional status of the study population. We also took into account the serum albumin levels and BMI of the study population. Previous studies have shown that there is an association between BMD and GNRI in patient populations with various diseases, including cardiovascular disease (O'Keefe et al., 2016), chronic obstructive pulmonary disease (Corsonello et al., 2015), and end-stage renal disease (Jeong et al., 2010). A systematic review reported that a low-protein diet was associated with hip fractures (Groenendijk et al., 2019). Higher protein intake may reduce BMD loss in the lumbar spine (Shams-White et al., 2017). In recent years, some scholars have also proposed that the GNRI can better predict short-term in-hospital mortality in elderly patients (Su et al., 2020) and mortality in patients on long-term haemodialysis or perit oneal dialysis (Valdez, 1991; Kang et al., 2013) and that patients with a GNRI >98 basically do not have adverse clinical outcomes. BMI and body weight can be used as indicators of energy intake, and the relationship between BMI and BMD has been described in many studies. The CI was proposed in the early 1990s as a measure of obesity and body fat distribution. The parameters used were body weight, height, and waist circumference (Valdez, 1991). The CI was based on the development of a biconical type of waist fat accumulation (Silva et al., 2013) and linked central obesity with cardiovascular disease. In recent years, studies have also shown that the CI can reflect the nutritional status of adolescents (Zhang et al., 2018) and patients on maintenance haemodialysis (Zhou et al., 2020) and can be used to predict the risk of cardiovascular events in elderly patients (Milagres et al., 2019). In this study, we compared the correlation between the GNRI, CI and BMD in the elderly population and found that there was a significant positive correlation between femoral neck BMD and GNRI in elderly patients (*p* < 0.001). However, a significant correlation was not found between BMD and CI in elderly patients. Although we did not find a correlation between albumin level and BMD in this study, because albumin level reflects protein status and is the main component of the GNRI, the effect of protein on bone may help to explain the relationship between the GNRI and BMD. Therefore, in combination with the findings of this study, the GNRI was closely related to bone mass and the incidence of OP in elderly patients. The nutritional status of elderly patients assessed by the GNRI was more closely correlated with BMD than that assessed by the CI and BMI, and the GNRI was even closely related to disease complications and patient survival rates.

In addition, in this study, it was found that maintaining an ideal body weight or having an actual body weight that slightly exceeds the ideal body weight was beneficial to maintaining better BMD in the elderly population, indirectly reflecting the nutritional status of the study population. Although there is an increasing number of elderly people, there are few reports that focus on their ADLs and 25-hydroxyvitamin D levels, especially for the elderly hospitalized population ((Nakamura et al., 2005; Houston et al., 2011; Kotlarczyk et al., 2017)). Another important finding of this study is that ADLs, 25-hydroxyvitamin D levels, BMD, and T-scores for elderly hospitalized patients were positively correlated in the multivariate regression analysis. The ADL scale mainly reflects the daily activity status of the study population and their own exercise capacity (Hannan et al., 2019), as well as their ability to perform various life skills in different contexts (Mlinac and Feng, 2016). Elderly people have poorer coordination and balance, and their response is slower than that of young people. Because they are often afraid of falling, some elderly individuals have a fear of exercising, thus aggravating the occurrence of OP and fractures (Hill et al., 2016). Although this study did not examine the muscle content in the study population, in some cases, such as when sedentary and r when fractures occur, the body is in a state of muscle unloading or activity immobilization, leading to a reduction in and atrophy of skeletal muscles (Houston et al., 2011).

25-Hydroxyvitamin D plays a major role in mineral metabolism and bone health through endocrine effects on bones, parathyroid glands, intestines and kidneys (Fleet, 2017). A low vitamin D status can lead to bone loss and low BMD. Some researchers have found that there are differences in vitamin D deficiency between races and regions, but there are few reports on the average vitamin D levels in elderly patients. The average level of 25-hydroxyvitamin D in elderly patients has been reported in Europe (Coin et al., 2008; Chen et al., 2019; Wang et al., 2020). In 2018, the UK scholar Marija found that the average 25-hydroxyvitamin D level in elderly patients with OP over 80 years old in a European community was 21.4 ng/mL (Hannan et al., 2019). Studies have also found that among 153 elderly OP patients in the UK, the median 25- hydroxyvitamin D level in women was 10.61 ng/mL (Coin et al., 2008), a concentration that was even lower than the lowest vitamin D level, 18.3 ng/mL in Belgian elderly women. Some scholars have also proposed that 25-hydroxyvitamin D levels are correlated with ADLs, to a certain extent. Researchers in Houston (Kotlarczyk et al., 2017) and Japan (Nakamura et al., 2005) propose that vitamin D deficiency is a predictive factor for low ADL scores and that women with low vitamin D levels have poorer physical function (Mlinac and Feng, 2016). The lower is the vitamin D level in elderly women, the greater is the decline in daily activity (Kotlarczyk et al., 2017). In this study, the 25-hydroxyvitamin D level in hospitalized elderly patients older than 60 years was 9.01 ± 5.51 ng/mL, and the proportion of elderly hospitalized patients with concentrations less than 20 ng/mL was as high as 96.8%. Furthermore, the ADL score for this group was 48.83 ± 25.64 points; both 25-hydroxyvitamin D and ADL score were risk factors for OP. Elderly hospitalized patients participate in a relatively small amount of outdoor activities, but severe vitamin D deficiency can cause osteomalacia, muscle atrophy and sarcopenia syndrome. Clinicians must pay more attention to the vitamin D level and exercise capacity of elderly patients and prevent OP and related complications in elderly patients. In summary, we propose that for elderly patients, good activity and 25-hydroxyvitamin D levels and good living conditions are conducive to the prevention of OP and the maintenance of high bone mass.

In this study, there were 20 patients with a history of fractures, and 16 patients were OP patients identified during the DXA BMD examination. Therefore, there is a close relationship between the occurrence of fractures and OP. For elderly patients, clinicians should also improve their awareness of OP in elderly patients, actively promote disease education and follow up, actively treat the disease and enhance fall assessments and education.

This study has several limitations. First, this is a cross-sectional study; Therefore, we cannot evaluate the longitudinal relationship between these parameters and BMD or T-scores and BMD. Further studies with larger samples sizes are required to validate the results. Second, we only evaluated hospitalized elderly patients older than 60 years of age and did not assess BMD in non-hospitalized elderly patients for comparison. In addition, although we believe that the presence of sarcopenia in elderly patients due to malnutrition and decreased exercise is even more pronounced in OP patients, the skeletal muscle mass of study patients was not evaluated. Most of the sample were women (*n* = 62), which makes it difficult to interpret/extrapolate the results for men. Further research is needed in other locations to evaluate other ethnic population. The importance of mood especially in hospitalized older adults would also be addressed in future study (Laudisio et al., 2018; Giovannini et al., 2019). Therefore, subsequent large-scale and more comprehensive studies are needed to investigate this hypothesis.

## 5 Conclusion

Much remains unknown about risk factors for OP in elderly and very elderly patients. In our study, patients at mean age of 83.56 years are enrolled. It is found that the BMD of the lumbar spine and femoral shaft in elderly patients significantly decreased with increasing age, and the incidence of OP significantly increased. In addition, BMD was closely related to sex, serum 25-hydroxyvitamin D level, the difference between actual body weight and ideal body weight, blood uric acid level, GNRI, and exercise capacity (ADL score). Focusing on the assessment of the nutritional status, exercise capacity, blood 25-hydroxyvitamin D level, and blood uric acid level in the elderly population can help to identify high-risk elderly patients with OP.

## Data Availability

The raw data supporting the conclusion of this article will be made available by the authors, without undue reservation.
